# Segmental forearm bone injuries in children: classification and treatment

**DOI:** 10.1007/s10195-015-0389-0

**Published:** 2016-02-09

**Authors:** N. K. Sferopoulos

**Affiliations:** Department of Pediatric Orthopaedics, Aristotle University of Thessaloniki, “G. Gennimatas” Hospital, P. Papageorgiou 3, 546 35 Thessaloníki, Greece

**Keywords:** Segmental injuries, Forearm bones, Children, Classification, Treatment

## Abstract

**Background:**

Fractures of the forearm bones in children are a very frequent injury, while segmental injuries of the forearm bones are very rare and have not been sufficiently examined. In this retrospective study, segmental injuries involving the radius, the ulna or both in children are classified and treatment outcome is presented.

**Materials and methods:**

Bone injury included any type of fracture or dislocation; segmental bone injury indicated the occurrence of more than one traumatic injury throughout the whole extent of each forearm bone. A total of 17 patients with 22 segmental bone injuries were identified and classified. Of these injuries, 12 involved the radius and 10 the ulna. The mean age at the time of injury was 8.9 years (range 3–13). In all cases, conservative treatment was the first treatment option; in three cases, however, surgical treatment was necessary.

**Results:**

All injuries were classified into five types using the new nomenclature. Patients were evaluated after an average follow-up of 10.4 years. Union was noted in all cases without any complications. The function results were rated as excellent in 15 cases and satisfactory in 2 cases.

**Conclusions:**

An inclusive classification system for segmental injuries of the forearm bones in children is presented. The proposed classification is a practical and utilitarian scheme that classified the patients of this report as well as all case reports previously published in the literature. It revealed that a wide variety of segmental injuries may be diagnosed following forearm injuries in children. This report also provided useful information that may influence the treatment of these complex injuries indicating that conservative treatment may be considered the first treatment option, and that primary surgical treatment is not justified.

**Level of evidence:**

Level V.

## Introduction

Although a fracture involving the forearm bones is the most common injury in childhood [1], little has been published about the incidence, classification and treatment of segmental injuries localized to either the radius or the ulna. The association of Monteggia or equivalent injuries with fractures of the distal part of the same forearm is the most frequently reported segmental injury [2–11], while the only previous attempt for classification of forearm segmental injuries in children was introduced by Sen et al. [12].

Seventeen children that were admitted for segmental injuries involving the radius, the ulna or both were included in the study. This report proposed a practical classification scheme, which was also tested on previously unclassified cases from the literature. It was also used to evaluate the final clinical and functional results following treatment of these complex bone injuries in children.

## Materials and methods

A total of 1377 children that were admitted for acute injuries of the radius and/or ulna between 1984 and 2013 were identified from the hospital database. Outpatient cases were not included in the study, since the radiographs of patients treated more than 2 years ago are usually recycled.

This search identified 17 children with segmental injuries involving the radius, the ulna or both. There were five patients with segmental injuries involving both forearm bones. There were 12 segmental injuries of the radius and 10 of the ulna. The average age of the 17 patients at injury was 8.9 years (range 3–13 years). There were 11 boys and 6 girls. All injuries resulted from a fall on the outstretched hand that occurred while running at sport or school. There were no polytrauma patients and injuries with vascular deficit or an acute compartment syndrome.

The radiographic examination typically included anteroposterior and lateral radiographs of the forearm, elbow and wrist. Computed tomography (CT) was used in only one patient.

**Fig. 1:**
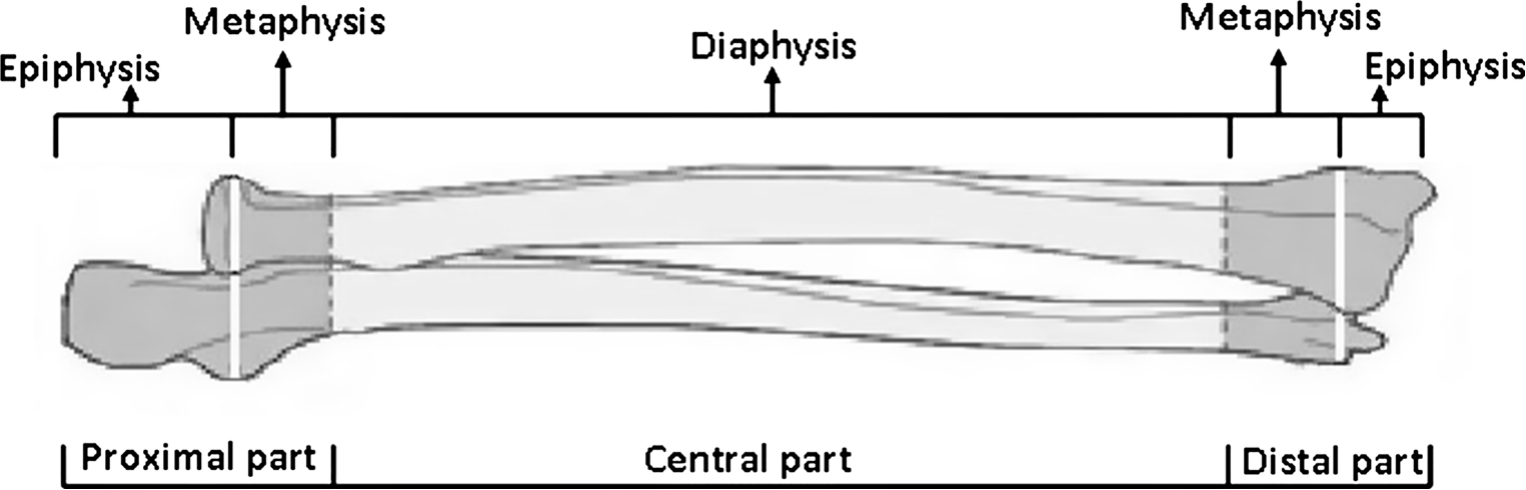
Schematic drawing of the division of the forearm bones in anatomical parts

Both radius and ulna were divided into three parts. The proximal part included the proximal epiphysis and metaphysis; the distal part included the distal epiphysis and metaphysis, while the central part included the diaphysis (Fig. 1). Bone injuries included fractures and dislocations, while fractures included all types of traumatic bone lesions such as complete or incomplete fractures, acute bowing or bone bruising. Segmental injuries indicated the appearance of more than one traumatic injury throughout the whole extent of each forearm bone. Segmental forearm bone injuries were classified in five types based on their location (Table 1). In type I lesion, injuries of the proximal and the distal part of the radius or the ulna were included; in type II lesion, injuries of the proximal part and the diaphysis were included; in type III lesion, injuries of the diaphysis and the distal part were included; in type IV lesion, bifocal injuries localized to a single part were included. Finally, in type V lesion, more than two traumatic injuries in each forearm bone were diagnosed.

**Table 1:** Classification system of segmental injuries of each forearm bone

Type	Traumatic bone injuries^a^	
	Number	Location (anatomical parts)
I	2	Proximal-distal
II	2	Proximal-central
III	2	Central-distal
IV	2	Same part
V	>2	Any part

^a^Traumatic bone injuries included any type of fracture or dislocation

In the patients of this report fractures of the proximal part of the forearm bones involved the proximal radial physeal plate or metaphysis, the olecranon or coronoid process, while dislocations included a dislocated elbow or a dislocated radial head. Lesions involving the central part included diaphyseal fractures. Finally, lesions involving the distal part included physeal or metaphyseal fractures of the radius or ulna.

Initial treatment was conservative in all cases. Open reduction and internal fixation was necessary following inadequate closed reduction in three patients suffering from: a fracture of the proximal radial epiphysis, a diaphyseal fracture of both forearm bones, and an open fracture of the distal radial metaphysis, respectively.

The patients were followed up for at least 1 year, and fracture union as well as forearm functional results were assessed according to the Anderson evaluation scale [13]. Final follow-up ranged from 1 to 29 years (average 10.4 years). The clinical and radiological data of these patients were reviewed retrospectively (Table 2).

**Table 2:** Clinical and radiographic evaluations

Patient no.	Gender/age		Radius/type		Ulna/type		Treatment	Follow-up (years)	Complications
1	M	8	fr/fr	I	fr	–	Combined	7	Limited pronation-supination (45 %)
2	M	11	fr	–	fr/fr	I	Combined	14	None
3	M	13	fr	–	fr/fr	I	Conservative	20	Limited elbow extension (15°)
4	M	12	fr	–	fr/fr	II	Combined	22	None
5	M	6	dl/fr	II	fr	–	Conservative	2	None
6	F	7	dl/fr	II	fr	–	Conservative	6	None
7	F	3	fr/fr	II	fr	–	Conservative	12	None
8	F	12	fr/fr	II	fr	–	Conservative	15	None
9	M	13	dl/fr	II	dl	–	Conservative	3	None
10	F	4	fr/fr	III	fr/fr	III	Conservative	3	None
11	M	4	dl/fr	I	fr/fr	III	Conservative	11	None
12	F	11	dl/fr	I	fr/fr	III	Conservative	1	None
13	M	7	fr/fr	III	fr/fr	IV	Conservative	29	None
14	M	6	fr	–	fr/fr	IV	Conservative	5	None
15	M	11	fr	–	fr/fr	IV	Conservative	25	None
16	M	13	fr/fr	IV	fr	–	Conservative	1	None
17	F	11	dl/fr/fr	V	fr/fr	III	Conservative	1	None

*fr* Fracture, *dl* dislocation

## Results

The radiological records, treatment and outcome were evaluated in each type of injury.

### Type I lesion

**Fig. 2:**
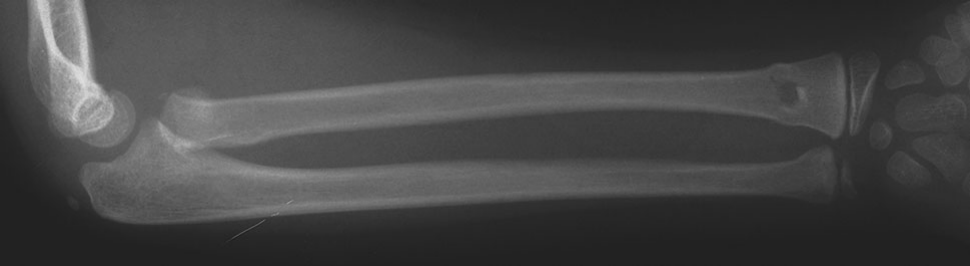
Radiograph of an 8-year-old boy with a type I segmental injury of the radius. It consisted of a displaced Salter-Harris type II physeal injury of the proximal radius associated with an undisplaced fracture of the distal part of the radius and ulna

**Fig. 3:**
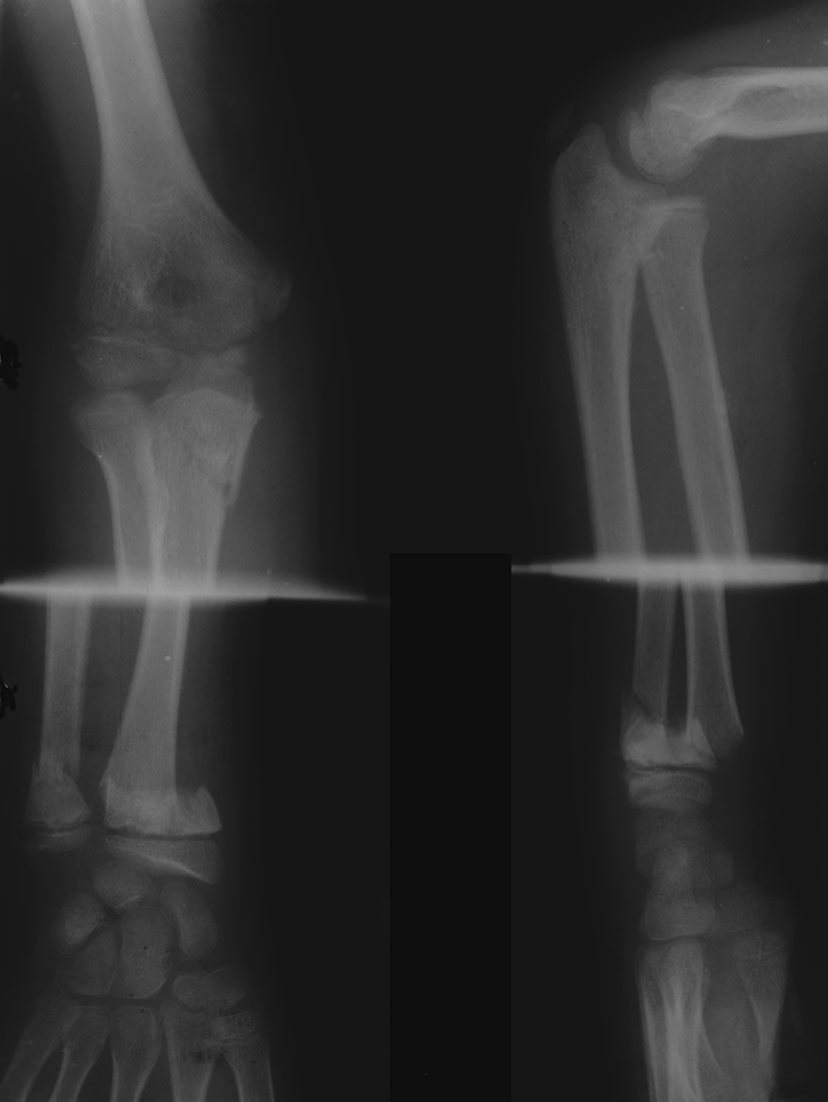
Radiographs of an 11-year-old boy with a type I segmental injury of the ulna. It presented as undisplaced fracture of the olecranon, and open displaced fractures of the distal part of the radius and ulna

Five injuries were identified; there were three segmental injuries of the radius and two segmental injuries of the ulna. The three segmental injuries of the radius included a fractured radial head in one patient (Fig. 2), and a dislocated radial head (Monteggia type I lesion) in two patients, respectively, associated with a distal physeal or metaphyseal fracture. The segmental injuries of the ulna included a fractured olecranon (Fig. 3) and coronoid process, respectively, associated with a distal metaphyseal fracture.

Four injuries were closed and one open with Gustilo grade-I severity. The fracture of the proximal radius (displaced Salter-Harris type II injury) was treated with open reduction and internal fixation with a pin through the lateral humeral condyle (Patient no. 1). Conservative treatment followed closed reduction in the Monteggia type I lesions, as well as in all remaining segmental injuries. In only one case (Patient no. 2) was the single distal radius fracture treated operatively (open injury). All fractures healed uneventfully; the results were graded as excellent on the Anderson scale, but two patients with a surgically treated fracture of the radial head and a conservatively treated fracture of the coronoid process of the ulna showed limited range of forearm pronation-supination (45 %) and loss of terminal extension (15°), respectively; the results were graded as satisfactory on the Anderson scale.

### Type II lesion

**Fig. 4:**
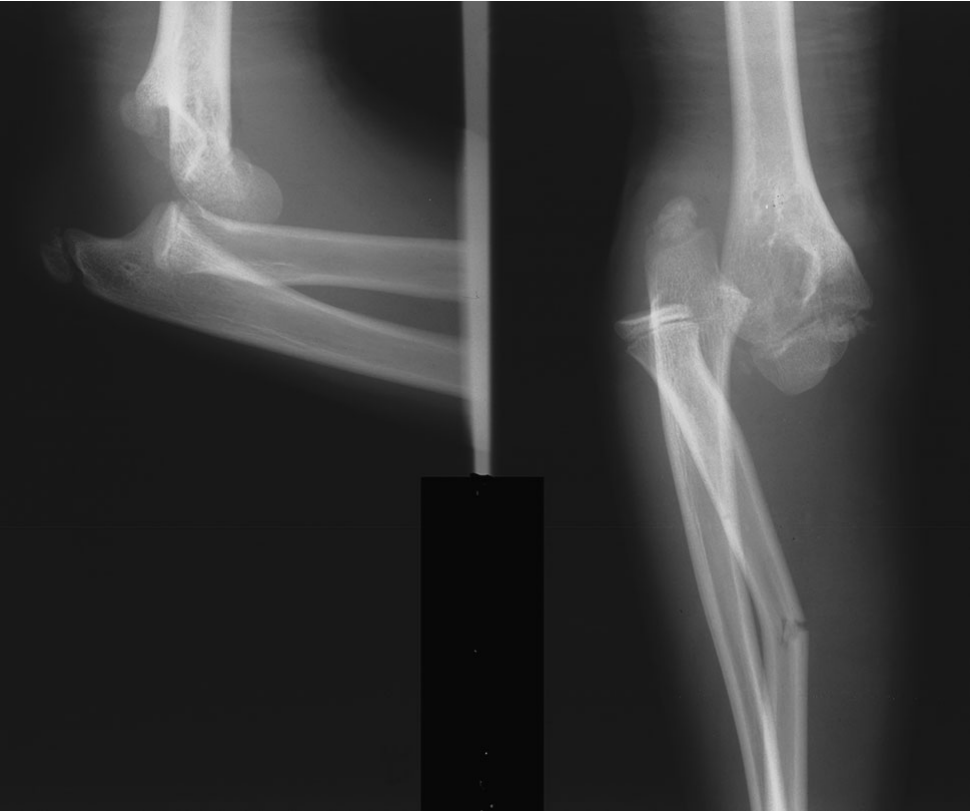
Radiographs of a 13-year-old boy with a type II segmental injury of the radius. It combined a dislocated elbow and a diaphyseal fracture of the radius

Six injuries were seen; there were five segmental injuries of the radius and one segmental injury of the ulna. Two patients were diagnosed with a Monteggia type IV injury. This consisted of a diaphyseal fracture of both radius and ulna associated with posterior and lateral dislocation of the radial head, respectively. Two patients appeared as a Monteggia type IV equivalent injury, i.e., a diaphyseal fracture of both radius and ulna associated with a physeal injury of the proximal radius. Both these cases have been published previously [14]. A dislocated elbow associated with a diaphyseal radial fracture was seen in one patient (Fig. 4). A diaphyseal fracture of both forearm bones associated with an olecranon fracture was diagnosed in one patient. None of the injuries was open. Conservative management followed closed reduction of the fractures of the proximal radius and ulna, as well as of the dislocated radial head and the dislocated elbow. Open reduction and internal fixation of the diaphyseal fractures of the radius and ulna with AO plates was performed in only one patient (Patient no. 4). All fractures united in normal alignment and the patients showed normal function of the elbow and wrist joints at follow-up; the results were graded as excellent on the Anderson scale.

### Type III lesion

**Fig. 5:**
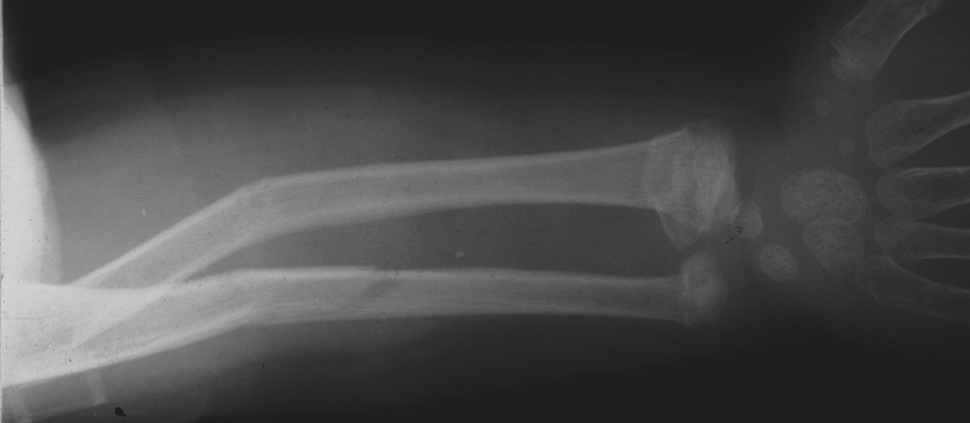
A 4-year-old girl with fractures of the diaphysis and the distal part of the radius and the ulna. A type III segmental injury of both the radius and the ulna was diagnosed

Six injuries were diagnosed; there were two segmental injuries of the radius and four segmental injuries of the ulna. In one patient a diaphyseal fracture of the radius and ulna was associated with a fracture of the distal part of both forearm bones (Fig. 5). A diaphyseal fracture of both forearm bones was associated with a distal metaphyseal fracture of the radius in another patient. Finally, a diaphyseal fracture of the ulna associated with a distal physeal fracture was diagnosed in three patients with a Monteggia type I injury. None of the injuries was open. All injuries were treated conservatively, united in normal alignment and the patients showed normal function of the elbow and wrist joints at follow-up; the results were graded as excellent on the Anderson scale.

### Type IV lesion

**Fig. 6:**
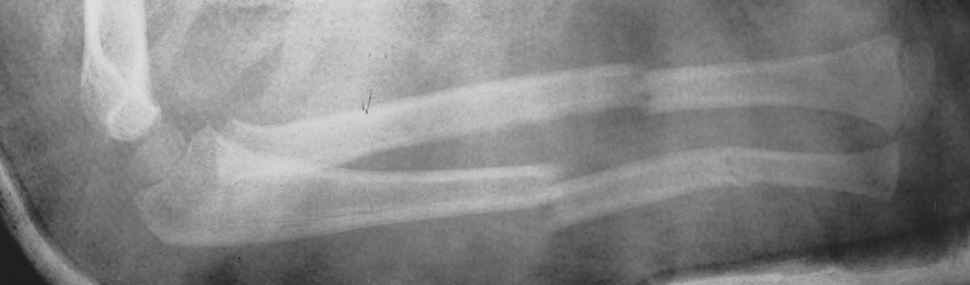
Radiograph of a 6-year-old boy with a type IV segmental injury of the ulna associated with a single diaphyseal fracture of the radius

Four bifocal diaphyseal fractures were detected: one to the radius and three to the ulna (Fig. 6). No bifocal injury localized to the proximal or distal part of the radius or the ulna was identified. Three injuries were closed and one open with Gustilo grade-I severity. All fractures were treated conservatively, united in normal alignment and the patients showed normal function of the elbow and wrist joints at follow-up; the results were graded as excellent on the Anderson scale.

### Type V lesion

**Fig. 7:**
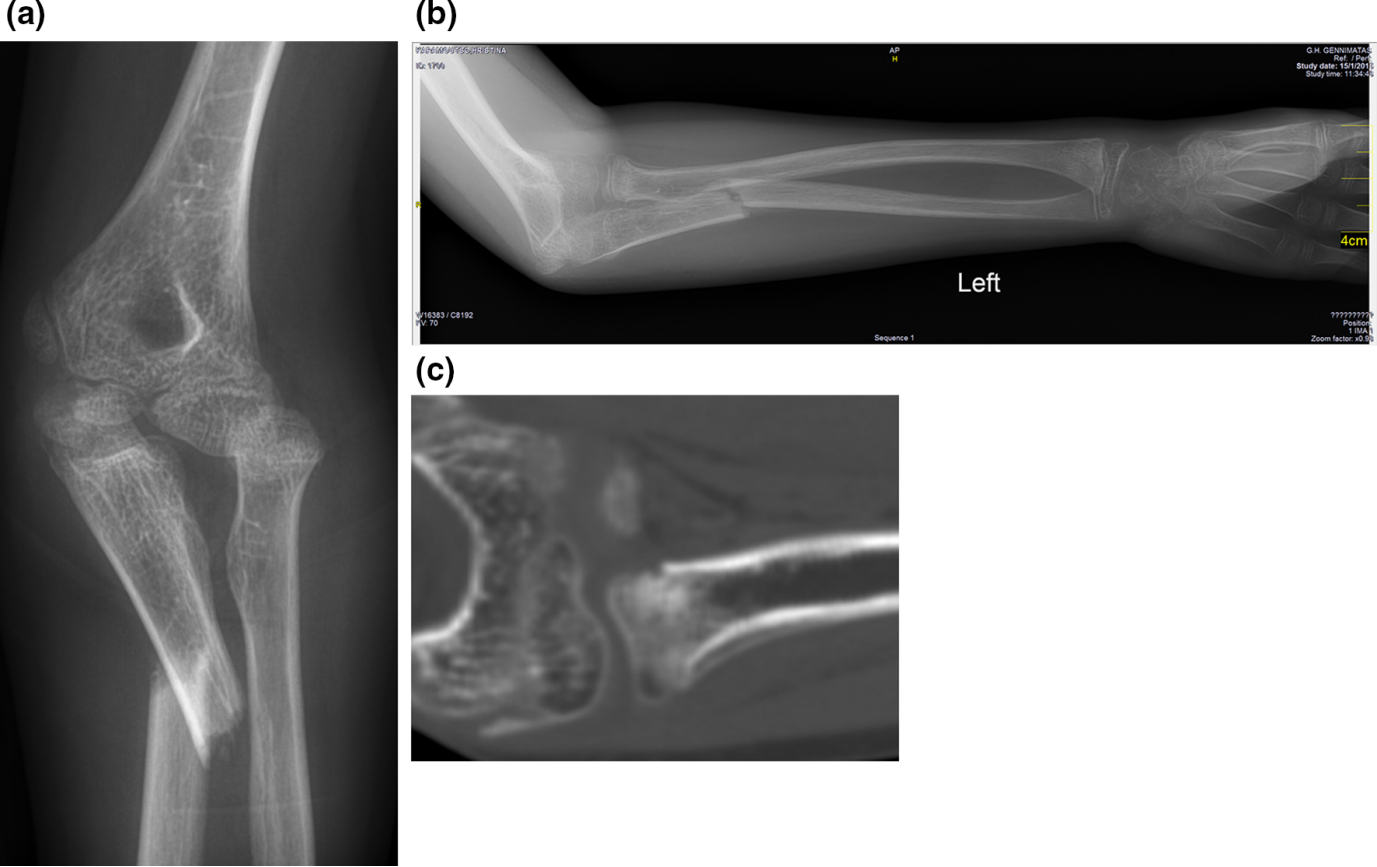
An 11-year-old girl with a lateral type Monteggia injury (**a**) associated with fractures of the distal part of the radius and the ulna. Radiographs at 5 weeks showed periosteal healing of the proximal radial metaphysis (**b**). Computed tomography (CT) indicated an additional fracture of the proximal radial metaphysis (**c**). A type V segmental injury of the radius and a type III segmental injury of the ulna were diagnosed

More than two traumatic injuries were diagnosed to the radius in one patient. Initial diagnosis indicated a lateral Monteggia injury (Fig. 7a) associated with distal radial and ulnar physeal fractures. Five weeks post-injury, following cast removal, signs of periosteal healing were evident at the proximal radial metaphysis (Fig. 7b). CT indicated an additional fracture of the proximal radial metaphysis (Fig. 7c). All injuries were treated conservatively, united in normal alignment and the patient showed normal function of the elbow and wrist joints at follow-up; the result was graded as excellent on the Anderson scale.

## Discussion

The use of classification schemes in pediatric orthopaedic trauma is a valuable aspect of description of fracture types for practice and research. It is also useful to evaluate the mechanism of injury as well as to guide management and treatment.

Using the only previously existing classification scheme introduced by Sen et al. [12], bifocal injuries of the radius in children were subdivided in two groups: group A included injuries such as Monteggia fractures or its variant associated with injuries of the distal forearm, and group B diaphyseal fractures associated with distal forearm injuries.

In the current study, a more inclusive classification scheme is proposed to label all various patterns of segmental forearm bone injuries in children taking into account apart from the radius, injuries of the ulna as well.

Type I lesion is consistent with the description proposed by Sen et al. [12] for group A injuries. All type I, II and III Monteggia injuries have occasionally been diagnosed to occur with ipsilateral fractures of the distal part of the forearm bones [2–6]. Monteggia equivalent injuries associated with distal forearm bone injuries have also been published [8–11]. In this report, fractures of the olecranon or the coronoid process associated with injuries of the distal part of the ulna were also included. The potential existence of a dislocated distal end of the ulna [15, 16] may also be encountered. Furthermore, the combined appearance of a dislocated elbow with a distal forearm injury that has already been described in the literature [17] may also be defined as a type I lesion.

In type II lesion Monteggia type IV injuries may be included. The appearance of a fractured radial head, instead, associated with diaphyseal forearm fractures has also been reported [14, 18] and it has been defined as a Monteggia type IV equivalent injury [14], which can also be included. Fractures of the olecranon or coronoid process associated with diaphyseal fractures of the ulna may also be included. A dislocated elbow associated with an ipsilateral diaphyseal fracture of the forearm bones, which has been once previously reported in the literature [19], was also included.

Type III lesion is consistent with the description proposed by Sen et al. [12] for group B injuries. This lesion has also been rarely presented in the literature [20, 21]. Although all cases of this report that were localized to the ulna included fractures, the existence of a dislocated distal end of the ulna may also be potentially encountered.

Type IV lesion injuries in this report were localized to the diaphysis. No cases with localization to the proximal or distal part of each forearm bone were identified.

Finally, type V lesion may include a Monteggia type IV lesion or an equivalent injury associated with a distal forearm injury [7] or other combined injuries like the one presented in this report.

Several terms have been used in the literature so far for the description of the occurrence of more than one traumatic injury throughout the whole extent of the same bone in children. These are described as bifocal, bipolar, double, segmental, multiple, multifragmentary, ipsilateral, simultaneous, combined or associated injuries. The term bifocal has been used for the classification of fractures of the tibia and fibula in adults [22]. The term segmental has been chosen in this report. Types I–IV lesions describe injuries that are double in origin, so they could be described as bifocal or bipolar. However, type V lesion includes more than two traumatic injuries of each forearm bone, so the term segmental was considered to be justified for the description of the whole spectrum of injuries. It indicated the occurrence of two or more traumatic injuries at the same or different parts of each forearm bone.

In our study, the rate of occurrence (1.2 %) was estimated among children admitted for fractures of the forearm bones. However, the true incidence would have been significantly lower, if undisplaced fractures, which do not usually necessitate admission, were taken into account.

Diagnosis is always based on the radiographic detection of the lesions. The elbow and wrist should always be included in the radiographic examination of forearm injuries. However, it is the clinical examination that will alert the trauma surgeon whenever an area of tenderness is revealed in a neighboring area where a secondary traumatic bone lesion may exist.

Failure to diagnose a radial head dislocation in children with midshaft forearm fractures has been recognized as a major complication [23]. In adults, the incidence of a missed dislocation has been reported to reach 42 % [24]. In addition, failure to diagnose a displaced fracture of the radial head is the major potential complication of a Monteggia type IV equivalent injury [14].

Segmental bone injuries in children are usually treated as separate injuries. Although, there are several generally accepted basic principles of treatment [25], parameters such as age [26] and an open injury [27] should be seriously considered. Furthermore, significant changes have been encountered in the operative treatment pattern. Closed reduction and percutaneous pinning rates have increased considerably to prevent the need for circumferential casting and reduce the risk of compartment syndrome [28]. However, conservative techniques still have a place in the treatment of pediatric forearm fractures with proper indications [29]. It is clearly evident that, as more information regarding these complex injuries becomes available, treating physicians are better equipped to diagnose and treat segmental forearm injuries in children. Since handling, sufficient traction and manipulation of the segmental forearm injuries that require reduction may not be easy for a single surgeon, we recommend referral to a pediatric orthopaedic department where one or two gentle attempts in the operating room under general anesthesia with muscle relaxation may be attempted. If closed reduction is unsuccessful or unstable, surgical intervention may be required.

This is the first attempt to describe a new reliable classification scheme of pediatric segmental forearm bone injuries. All previously described series and case reports as well as the patients of this study could be classified in the described five types of segmental forearm bone injuries. All included types of injury are precise and can be diagnosed on radiographs easily even by the non-experienced trauma surgeon. However, its greatest importance is to reveal that a wide variety of segmental injuries may be diagnosed following forearm injuries in children, and to indicate the role of clinical examination as the first and most important step towards making a definitive diagnosis.

In addition, the treatment outcome of the patients in this report may influence treatment of these complex injuries. Clinical and functional results following treatment clearly indicated that conservative modalities should always be considered as the first treatment option and that primary surgical treatment is not justified. Finally, epidemiological studies of segmental injuries with localization other than the forearm bones will be required to show whether the reported classification system could be generally applicable to all tubular bones of the immature skeleton and, furthermore, whether it could also be applied to adult patients.
